# Smart aggregation‐induced emission polymers: Preparation, properties and bio‐applications

**DOI:** 10.1002/smo.20220008

**Published:** 2023-03-27

**Authors:** Guiquan Zhang, Xinyao Fu, Daming Zhou, Rong Hu, Anjun Qin, Ben Zhong Tang

**Affiliations:** ^1^ State Key Laboratory of Luminescent Materials and Devices Guangdong Provincial Key Laboratory of Luminescence from Molecular Aggregates Center for Aggregation‐Induced Emission AIE Institute South China University of Technology Guangzhou China; ^2^ School of Chemistry and Chemical Engineering University of South China Hengyang China; ^3^ Center for Aggregation‐Induced Emission AIE Institute South China University of Technology Guangzhou China; ^4^ School of Science and Engineering Shenzhen Institute of Aggregate Science and Technology The Chinese University of Hong Kong Shenzhen China; ^5^ Hong Kong Branch of Chinese National Engineering Research Centre for Tissue Restoration and Reconstruction The Hong Kong University of Science & Technology Hong Kong China

**Keywords:** aggregation‐induced emission, lab‐in‐cell, multifunctional integration, smart polymer, synergistic amplification

## Abstract

Owing to the outstanding photophysical properties, organic luminescent materials featuring aggregation‐induced emission (AIE) characteristics have attracted wide attention in various fields. Numerous researches focused on low‐mass AIE luminogens, and relatively less attention has been paid on AIE polymers and the related applications, in spite of the fact that AIE polymers exhibit excellent advantages of processability, multifunctional integration and synergistic effects. In this review, we briefly summarize and discuss the superiorities of AIE polymers in preparation, properties and bio‐applications, and the considerable progress in these aspects are introduced as well. Finally, the structure‐property relationship, challenges and opportunities are also discussed. Hopefully, this review will be a trigger for smart AIE polymer research and further broaden their applications.

## Graphical abstract

In this review, we briefly summarized the preparation, properties and applications of smart aggregation‐induced emission (AIE) polymers in recent years with the emphasis on the advantages of AIE polymers over theri low‐mass analogs and some related commercially available low‐mass molecules. Meanwhile, the challenges and opportunity in this area are also briefly discussed.

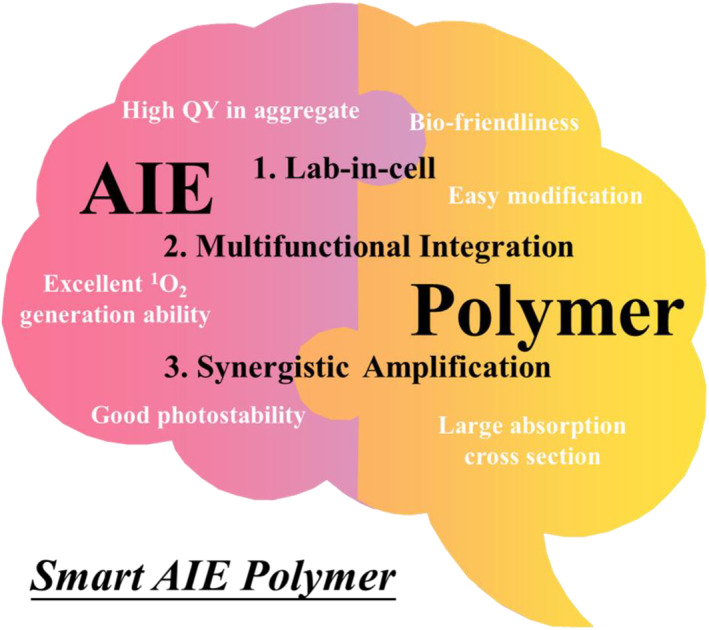

## 1 INTRODUCTION

Organic luminescent materials present significant potential in the real‐time monitoring of bio‐analytes and dynamic processes in living organisms, benefited from the advantages of good biocompatibility, property‐structure tunability and preparation repeatability.^[1]^ In recent years, kinds of organic fluorescent materials have been developed for bio‐imaging, diagnosis and therapeutic applications with good performances.[^2^, ^3^, ^4^, ^5^] Conventional fluorescent materials with conjugated structures display bright luminescence in the molecular state. However, these molecules will rapidly form aggregates and cause fluorescence quenching due to the strong intermolecular interactions under physiological environment, which is known as aggregation‐caused quenching (ACQ) effect. This phenomenon seriously reduces the sensitivity and further limits the biological applications of organic luminescent materials.^[6]^ In 2001, Tang and co‐workers observed a new photophysical phenomenon which was coined as aggregation‐induced emission (AIE).^[7]^ Opposing to ACQ materials, the luminogens with AIE properties (AIEgens) exhibit bright fluorescence in the aggregate state by restricting intramolecular motion. Based on this guideline, more and more molecules with AIE feature are designed and widely used in various applications.^[8]^ Excitingly, the rapid development of AIE research has provided the foundation for the aggregate science.^[9]^

AIE polymers, an emerging research discipline, is expected to be ideal materials for diverse applications attributed to the promising properties. By introducing AIE units to the polymers, the resultant AIE polymers will incorporate the advantages of both AIEgens and polymers, showing excellent biologic application potential. Nevertheless, the advantages of AIE polymers over low‐mass AIEgens need to be further complemented and summarized. In this review, we will introduce and discuss the AIE polymers with specific properties for biological applications, namely smart AIE polymers. Meanwhile, biopreparation approaches and two important properties of multifunctional integration and synergistic effect for AIE polymers will be introduced and summarized in detail. Moreover, the perspective of the future development direction and challenges for smart AIE polymers are briefly discussed.

## 2 PREPARATION AND FEATURES OF NOVEL SMART AIE POLYMER

According to the position of AIE units in the polymer, AIE polymers can be classified as main‐chain and side‐chain types. Thanks to the advantages of AIEgens and polymeric materials, the smart AIE polymers have achieved considerable progress in the preparation of biomacromolecules and applications in biosensing, imaging and therapeutics fields. In 2003, the first AIE polymer was prepared by introducing 1,2,3,4,5‐pentaphenylsilolyl (a typical AIEgen) as the pendant of polyacetylene, which opened the door to the research of AIE polymers.^[10]^ With the participation of metal‐containing catalysts NbCl_5_‐ and WCl_6_‐Ph_4_Sn, the resultant AIE polymers exhibited good solubility, high molecular weights and good thermal stability. Thanks for the existence of AIE units, the efficiency of polyacetylene‐based electroluminescence devices was further improved. Motivated by this work, more and more AIE polymers with novel structures and functions were synthesized through diverse routes, including Suzuki, Sonogashira, Stille, Mcmurry polymerizations and so on.^[11]^ These AIE polymers have displayed promising applications in various fields, such as bioimaging, diagnostic and therapeutic ones. However, the metal‐containing catalysts and strict reaction conditions during the polymerization limit the biological applications of the resultant AIE polymers owing to the cytotoxicity of metal ions.

### 2.1 Lab in cell

The biochemical process, operating all the time in vivo, is directly related to the metabolic functions of organisms. By introducing the unnatural functional groups, drugs and reactive moiety into biomolecules and cells, utilizing the natural or unnatural reactions inside cells have become a powerful method for biological studies. For this reason, synthesizing smart AIE polymer *in situ* has attracted increasing attention to acquire desirable function and applications.

**FIGURE 1:**
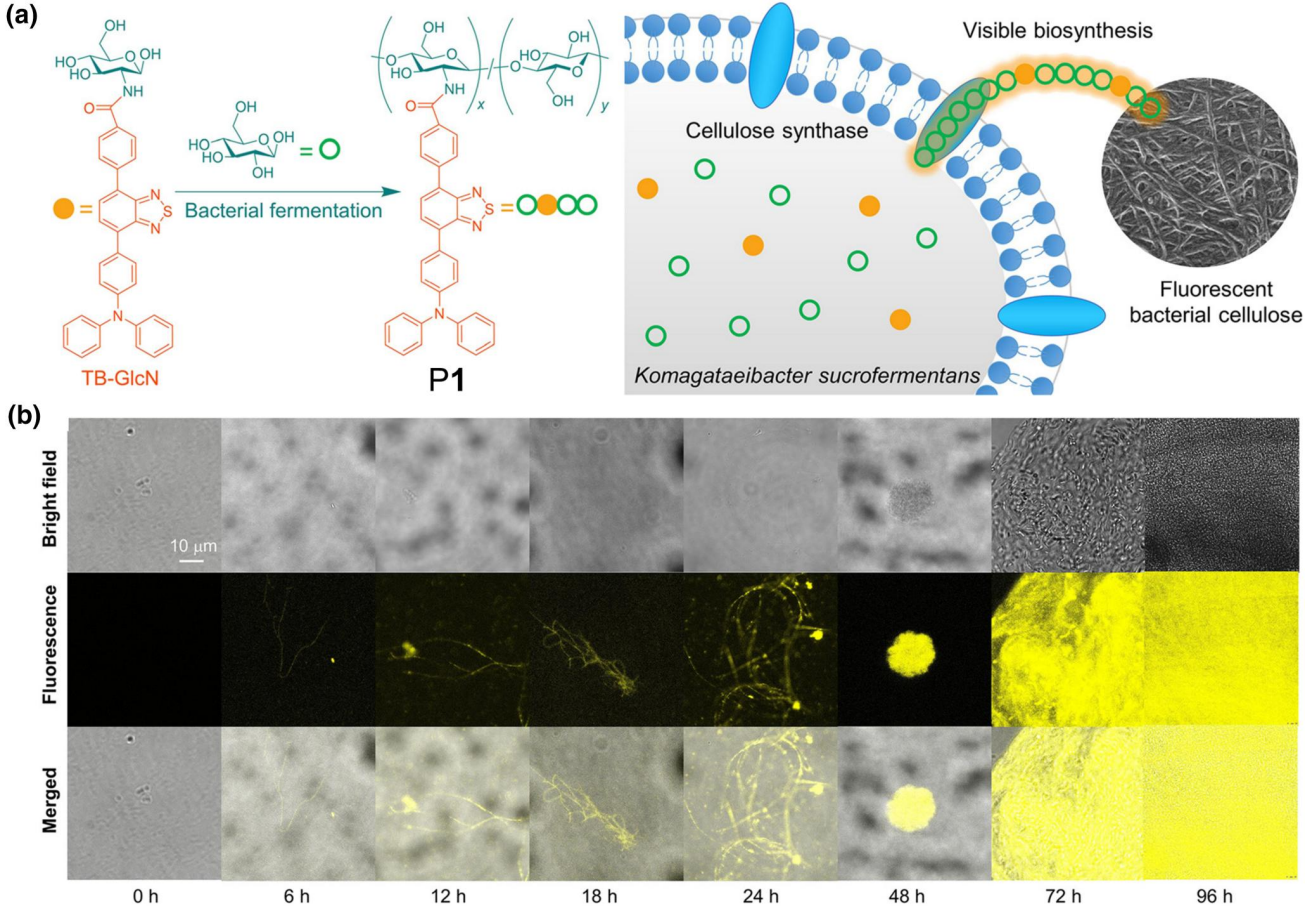
(a) Structure of AIEgen‐glucosamine, AIE polymer P**1** and schematic diagram of biological synthesis of fluorescent cellulose. (b) CLSM images of P**1** through bacterial fermentation. Reproduced with permission: Copyright 2022, American Chemical Society.^[12]^ AIE, aggregation‐induced emission; CLSM, confocal laser scanning microscope.

Visualization of biological process is one of the most direct and rapid methods to explore and understand life. In 2022, Tang, Han and co‐workers designed and synthesized a low‐mass AIEgen‐functionalized glucosamine to visualize the bacterial fermentation process (Figure 1a).^[12]^ Triphenylamine and benzothiadiazole were selected as electron‐donating and withdrawing group, respectively, to construct the high‐bright yellow emissive AIEgens, which allowed the visualization of the cellulose formation process and avoided the fluorescence quenching in the solid state due to the π‐π stacking. The bright fluorescence could light up the early microfiber, which was difficult to be observed by naked eyes (Figure 1b). Meanwhile, the brightness of fluorescent cellulose P**1** could be adjusted by regulating the concentration of AIEgen‐glucosamine. Compared with cellulose adsorbed with fluorescent molecules, the biosynthetic fluorescent cellulose exhibited superior properties which made it more desirable for further applications.

The introduction of AIE units allows not only the visualization of biological processes, but also realization of specific functions. Liu and co‐workers constructed an AIE bacterial cellulose in a similar way.^[13]^ The introduction of AIE units furnished the polymers with reactive oxygen species (ROS) generation ability under light irradiation, achieving light‐controlled sterilization and promoting the rapid wound healing. The preparation strategy based on biochemical process of organism provides important insights for the synthesis and dynamic process of the related biomaterials.

**FIGURE 2:**
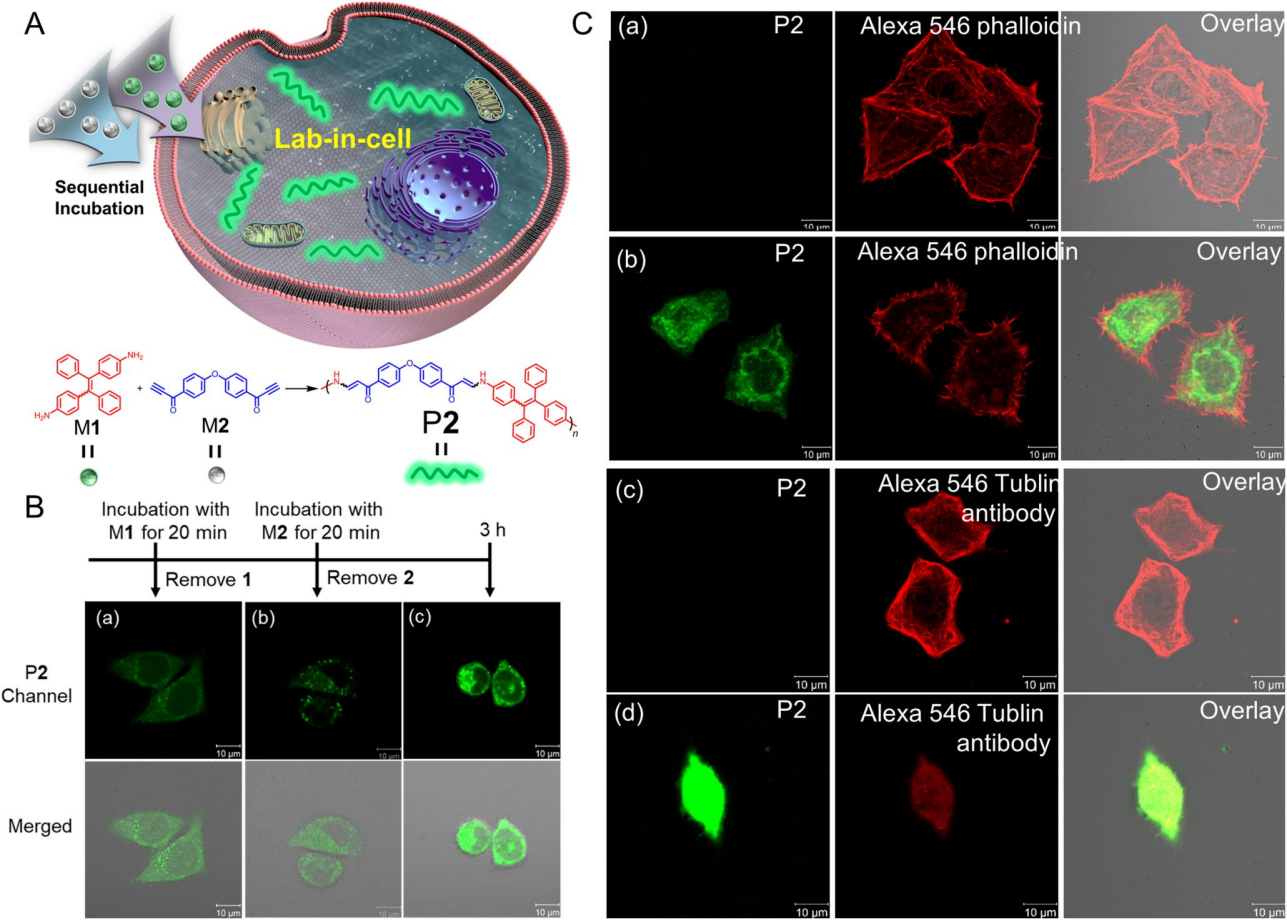
(A) Schematic diagram of intracellular spontaneous amino−yne click polymerization and synthesis method of P**2**. (B) CLSM images of HeLa cells after incubated with M**1** (a), M**2** (b) and the long‐term incubation (c). (C) Mechanism of cell death caused by intracellular polymerization. CLSM images of HeLa cells without (a, c) and with (b, d) intracellular polymerization, then marked with Alexa 546 phalloidin or Alexa 546 Tubulin antibody. Reproduced with permission: Copyright 2019, Springer Nature.^[15]^ CLSM, confocal laser scanning microscope.

Compared to post‐modification of natural macromolecules or low‐mass precursors, it is more straightforward and efficient to artificially introduce reactions that can produce AIE polymers *in situ* under physiological conditions for the diagnosis and therapy applications. In addition, such approaches are attracting increasing attention and present high potential for biomedical applications. In 2019, with the help of the spontaneous amino‐yne click polymerization,^[14]^ Qin, Tang and co‐workers developed an *in situ* strategy to synthesize AIE polymers in living cells and achieved “turn‐on” fluorescent imaging and anti‐tumor effects.^[15]^ As shown in Figure 2A, owing to the high efficiency and spontaneity of the reaction, a polymer P**2** with a molecular weight of 7300 could be facilely obtained by incubating AIEgen‐containing diamine M**1** and carbonyl group‐activated terminal diyne M**2** with cells for 20 min, respectively. Interestingly, this polymerization is more efficient in water than in organic solvent, indicating potential applications in biomedicine. Furthermore, it was worth noting that the “turn‐on” fluorescence of the cells resulted from the polymerization reaction could not be achieved by co‐incubation of cells with M**1**, M**2** or P**2** prepared *in vitro*, respectively (Figure 2B). At the end of the intracellular polymerization, the generated polymer could destroy the cytoskeleton, including tubulin and actin, leading to *in situ* killing of tumor cells (Figure 2C). This work broadens the application of spontaneous amino‐yne click polymerizations in biomedical science and provides new insights into the imaging and drug‐free therapy of cancer.

**FIGURE 3:**
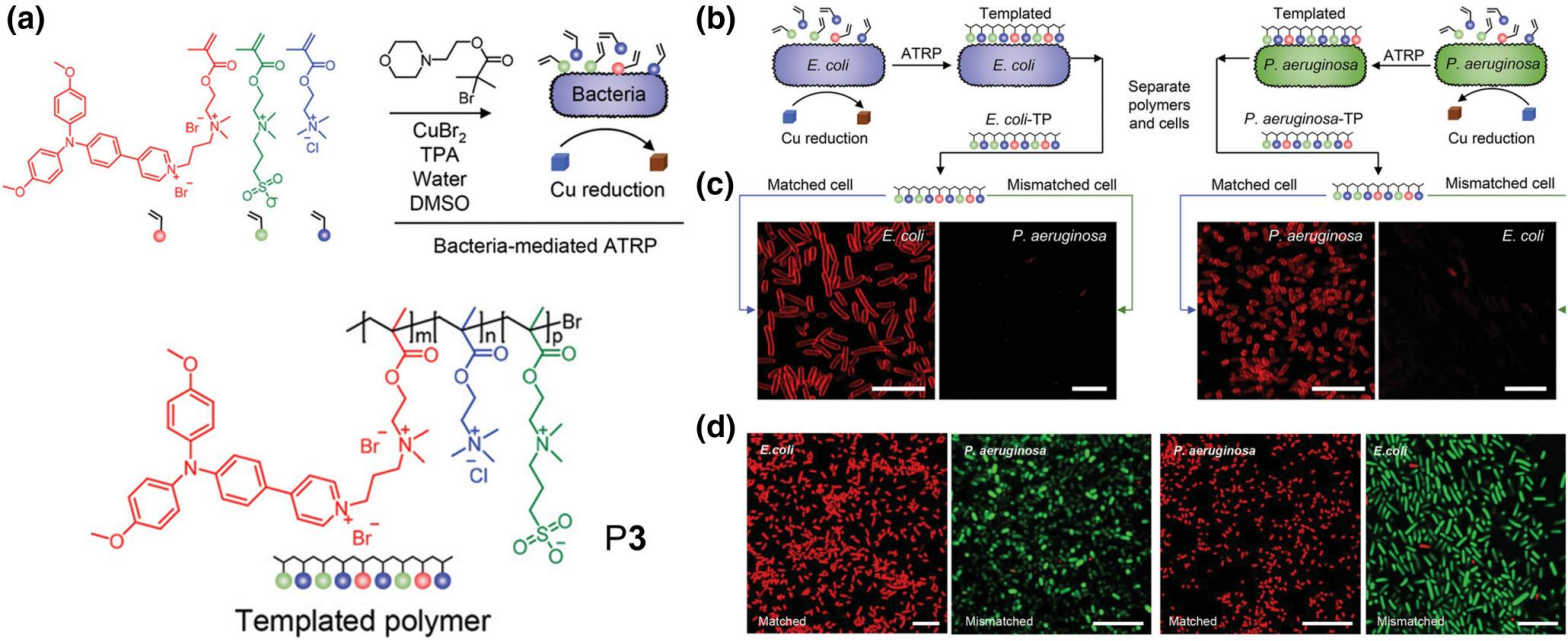
(a) Schematic diagram of the bacteria‐mediated templating process. (b) Schematic diagram of template polymer P**3** synthesis. *E. coli* and *P. aeruginosa* were selected as templates for the direct synthesis of template polymers, and the synthesized template polymers were obtained by washing with 0.15 M NaCl solution. (c) Confocal laser scanning microscope images of *E. coli* and *P. aeruginosa* after incubation with matched polymers and mismatched polymers at the concentration of 600 ng mL^−1^. (d) Live/dead bacterial viability assay of *E. coli* and *P. aeruginosa* after incubation with their templated polymers and mismatched polymers with light irradiation for 20 min. Reproduced with permission: Copyright 2020, John Wiley and Sons.^[16]^

Currently, the overuse of broad‐spectrum antibiotics has led to the emergence of a large number of drug‐resistant pathogens, which has caused great concern. Researchers have made a worthwhile effort to recognize pathogenic microorganisms and inactivate them quickly and accurately. However, there are still some problems need to be solved, one of the most critical issues is the specific identification of pathogenic microorganisms. To solve this problem, Liu and co‐workers proposed an *in situ* strategy to synthesize smart AIE polymers and achieve selective antibacterial activity by using specific bacteria as a template.^[16]^ As presented in Figure 3a, using a matched bacteria template, AIE polymer P**3** was prepared by atom transfer radical polymerization (ATRP). The selection of monomers by the template bacteria directly affected the structure of the templated AIE polymer (Figure 3b), and bacterial fluorescence “turn‐on” must be depended on the matching of the polymer to the template bacteria (Figure 3c). Moreover, when exposed to light irradiation, the AIE unit inserted in the polymer P**3** as side chain could generate ROS and achieve selective inactivation of the template bacteria. However, due to the weak binding interaction between templated polymers and non‐templated bacteria, poor antibacterial effect was observed even under light conditions (Figure 3d). This strategy provides a new approach for the specific recognition and treatment of pathogenic microorganisms. Meanwhile, it also shows that *in situ* construction of AIE polymers has high potential in biomedical science.

### 2.2 Multifunctional integration

The rapidly developed synthetic strategies and structurally ingenious design promise to obtain smart AIE polymers with unique properties. Up to now, AIE polymers have made remarkable progress in several areas such as biosensing, bioimaging and diagnostics.^[17]^ Tian and co‐workers introduced fluorine‐containing motifs and AIEgen into amphiphilic polymers by side chain modification in 2012.^[18]^ Then the resultant polymers with high fluorescence quantum yields were successfully used for cytoplasmic imaging. Lu, Xu and co‐workers prepared the polysulfates bearing AIE units and naphthylamide groups using a sulfur(VI) fluoride exchange (SuFEx) click reaction.^[19]^ Owing to the existence of the naphthylamide groups, the obtained AIE polymers could achieve selective detection of cyanide ions with much lower detection limits than those reported low‐mass molecules and non‐AIE polymers so far. Different from the normal cells, lipid droplets are more numerous and less polarized in cancer cells. Lin and co‐workers achieved the selective imaging of lipid droplets in cancer cells by introducing the AIE unit with a polarity‐responsive D‐π‐A structure into biocompatible polysiloxane.^[20]^

As the research progressed, scientists realized that the advantages of AIE polymers could not be fully exploited by relying solely on the characteristics of the introduced AIE units. Therefore, in recent years, researchers have worked on the preparation of innovative smart AIE polymers, which combines the inherent properties of polymers with AIE effect to achieve a win‐win strategy.

**FIGURE 4:**
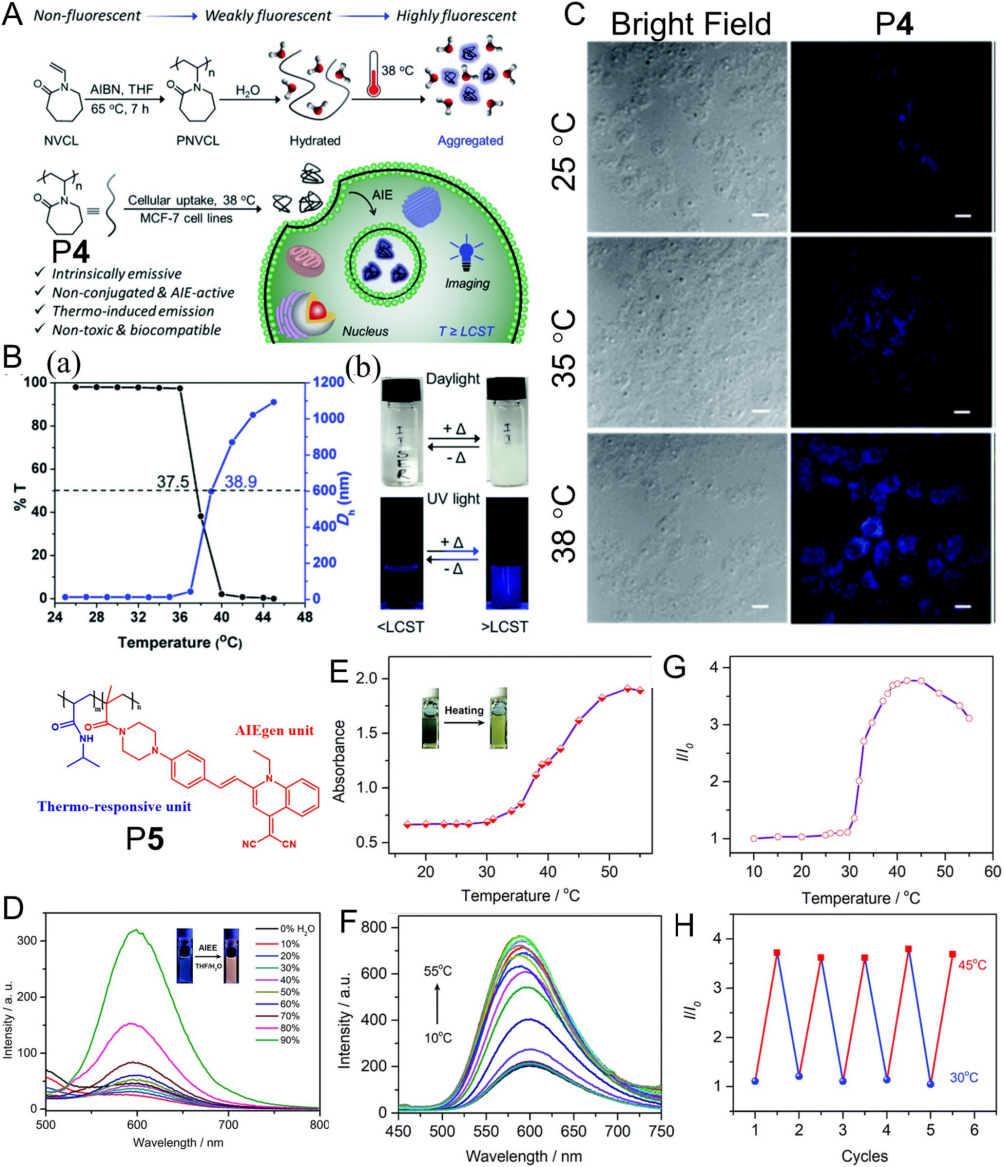
(A) Synthesis route of polymer P**4** and schematic diagram of intracellular temperature imaging of P**4** as a fluorescent thermometer. (B) Variation of transmittance (*T*) and average hydrodynamic diameters (*D*_h_) of P**4** aqueous solution as a function of temperature (a). Photographs of P**4** solution obtained below and above the LCST under different irradiation conditions (daylight and 365 nm) (b). (C) CLSM images of MCF‐7 cells after incubation with polymer P**4** (250 μg mL^−1^) at 25, 35, 38°C for 24 h. Reproduced with permission: Copyright 2020, Royal Society of Chemistry.^[21]^ (D) AEE behaviors of the P**5**. (E–H) LCST‐featured behaviors of copolymer P**5**: temperature‐dependent absorption (E), emission (F), relative fluorescent intensity (G) and thermo‐response reversibility (H). Reproduced with permission: Copyright 2019, Royal Society of Chemistry.^[22]^ AEE, aggregation‐enhanced emission; CLSM, confocal laser scanning microscope; LCST, lower critical solution temperature.

Temperature is closely associated with biochemical reactions and biological processes, such as ATP hydrolysis and hyperpyrexia, which is one of the important physiological parameters of cells. However, when cells become dysfunctional or abnormal, for example, cancer or inflammation, the temperature of the cells will be changed. Therefore, the development of highly accurate thermometers which can monitor the temperature change in real time will help analyze the cell status and give a reasonable treatment plan in time. In 2020, De and co‐workers fabricated an elegant AIE non‐conjugated poly(*N*‐vinylcaprolactam) (PNVCL) P**4** with significant temperature‐induced phase transition.^[21]^ As shown in Figure 4A, P**4** could be obtained by a free radical polymerization. Compared with the reported low‐mass molecules and polymeric thermometers, the synthesis of P**4** was relatively simple and could be obtained in only one step. Meanwhile, the unsaturated C=O functional groups contained in the branched chains provided P**4** with non‐conventional luminescent properties, which was known as clusteroluminescence. It was noteworthy noting that the photoluminescence (PL) intensity of the polymer solution significantly enhanced when the poor solvent of hexane was gradually added to the system. With the increase of the poor solvent, the PL intensity of the solution could be enhanced up to 5‐fold, which meant that P**4** had a significant AIE property.

In addition, the good water solubility and temperature‐dependent phase transition behavior of PNVCL were verified as important conditions for becoming fluorescent thermometers (Figure 4B). When the temperature was below the lower critical solution temperature (LCST), P**4** could dissolve well in water due to the hydrogen bonding between ‐OH groups of water molecules and C=O groups on the lactam. Then the rupture of hydrogen bonds with increasing temperature was an important reason for the significant decrease in the solubility of P**4** in water. Such a process led to the formation of P**4** aggregates, which resulted in the turn‐on of fluorescence. Based on this, the monitoring of intracellular temperature could be achieved after co‐incubation of P**4** with MCF‐7 cells. After co‐incubation at different temperatures for 24 h, a large difference of fluorescent intensity between different groups was observed (Figure 4C). For cells, different temperatures may have different cellular uptake capacity, which may result in different intracellular fluorescence intensities. Indeed, this also indirectly reflects the importance of temperature on cellular biological activities. The simple preparation, inherent clusteroluminescent properties and long‐lasting trace properties derived from the polymer give these smart AIE polymers a wide range of applications.

The preparation of fluorescent thermometers using thermally responsive non‐traditional AIE polymers is a promising strategy. However, their inherent properties of short absorption and emission wavelengths and low fluorescence quantum yields limit their further application as fluorescence thermometers. Zhu and co‐workers obtained poly(*N*‐isopropylacrylamide) P**5** by combining the thermal‐responsive monomer *N*‐isopropyl acrylamide and AIE unit via free radical polymerization.^[22]^ The obtained polymer P**5** had significant AIE property, which was beneficial for intracellular temperature imaging (Figure 4D). With an increase of temperature, the absorption and emission of the polymer in aqueous solution significantly enhanced (Figure 4E–G), indicative of its good temperature response property. Temperature cycling experiments confirmed that P**5** had good stability (Figure 4H), which made it more superior in the field of biological temperature sensing.

**FIGURE 5:**
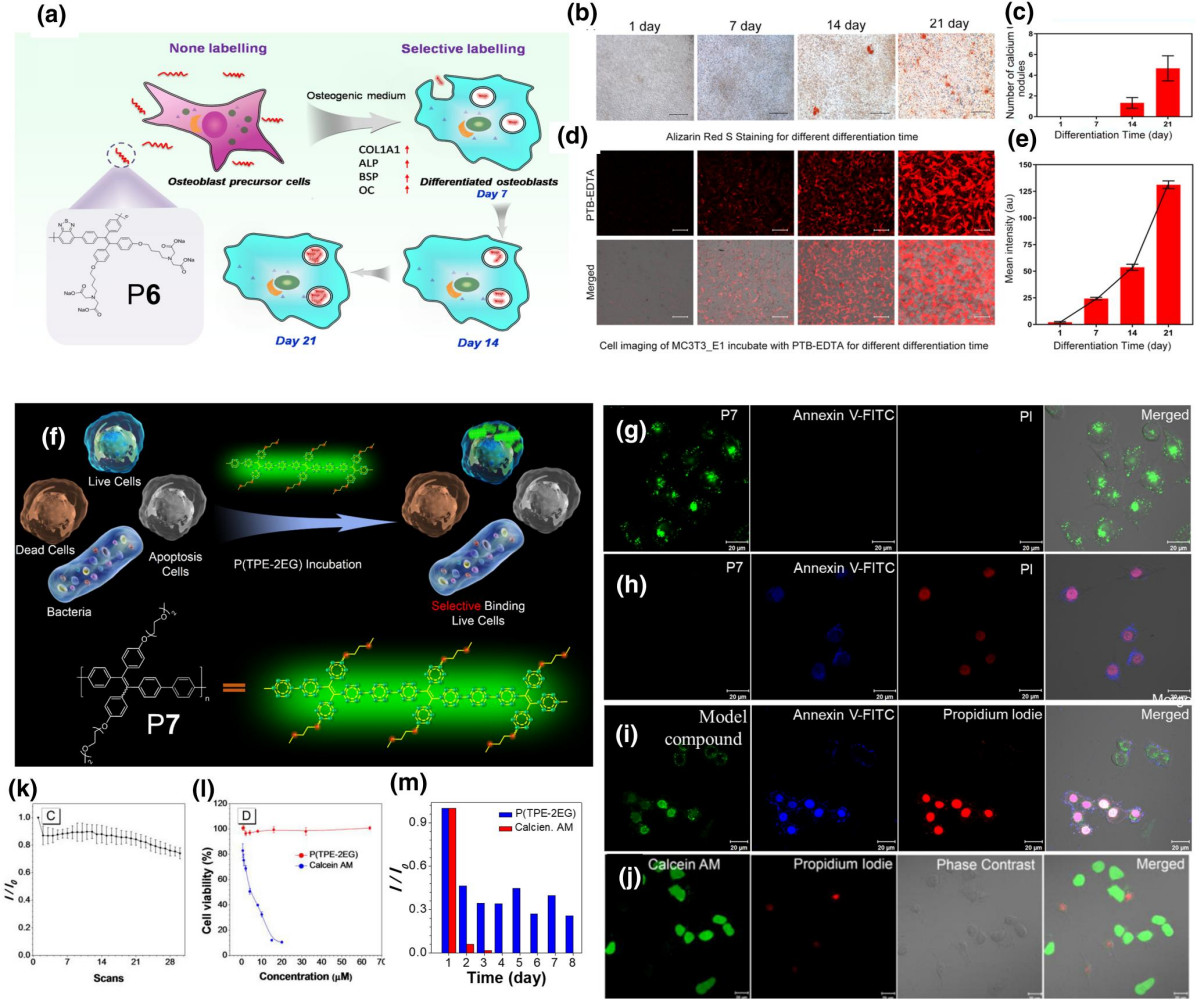
(a) Chemical structure of P**6** and schematic diagram of the cellular internalization and monitoring of osteogenic differentiation in real time. (b–e) CLSM images and fluorescence statistics of MC3T3‐E1 cells after incubation with Alizarin Red S (b, c) or polymer P**6** (d, e) at the indicated differentiation times. Reproduced with permission: Copyright 2020, Elsevier.^[23]^ (f) Schematic diagram of polymer P**7** selectively differentiating living cells from apoptotic cells, dead cells and bacteria. (g–j) CLSM images of HeLa cells after incubated with polymer P**7** (g, h), model compound (i) and Calcein AM (j), respectively. (g–j) HeLa cells treated with 0, 1000, 500, 500 μM H_2_O_2_ solution. (k) Photostability of P**7** under laser irradiation (405 nm). (l) Cell viability of HeLa cells incubated with P**7** and Calcein AM at different concentrations. (m) Histogram of the fluorescence intensity changes after incubation of P**7** and Calcien AM with HeLa cells. Reproduced with permission: Copyright 2020, Elsevier.^[24]^ CLSM, confocal laser scanning microscope.

The bright emission and favorable conjugation of smart AIE polymers are not only used in biosensing, but also in highly sensitive bioimaging. Osteogenic differentiation is a complex process which contains large amounts of important information. Monitoring the osteogenic differentiation process accurately and rapidly is essential for the assessment of related diseases. In 2020, Zhang, Qin and co‐workers prepared a smart AIE polymer P**6** that could selectively chelate Ca^2+^ (Figure 5a).^[23]^ P**6** consistes of a conjugated main chain and an ethylenediaminetetraacetic acid (EDTA) side chain. The conjugated structure containing AIE‐active tetraphenylthene and benzothiadiazole offers P**6** with outstanding emission and long‐term tracing capability. EDTA enhanced the solubility of P**6** and endowed it with high Ca^2+^ coordination, which was the basis for the monitoring of osteogenic differentiation. After efficient chelation with Ca^2+^, P**6** crossed the cell membrane by endocytosis to achieve long‐term tracing of osteogenic differentiation cells, which was not found with its low‐mass model compounds. Notably, the P**6**‐based osteogenic differentiation detection was much more sensitive than Alizarin Red S staining. As shown in Figure 5b–e, after 7 days of cocultivation of P**6** with MC3T3‐E1, an osteogenic differentiated cell, clear fluorescent signal could be found in cells. However, the signal of Alizarin Red S became visible after 14 days of staining.

Efficient and rapid monitoring the cell viability of cancer cells can provide key information about the therapeutic effects of cancer. Qin, Tang and co‐workers have successfully designed a smart AIE conjugated polymer P**7**, which could selectively image live cells (Figure 5f).^[24]^ For bioimaging, the side chains of oligo(ethylene glycol)s with negative charge reduced the non‐specific interaction between P**7** and cell membrane, which made it obligatory to enter the cell via endocytosis (Figure 5g). On the other hand, the model compound showed no selectivity due to its low molecular weight, which allowed entering and leaving cells by free diffusion (Figure 5i). This result indicates that polymer has a distinct advantage in selective imaging. Compared with lower‐mass organic molecule probe (Calcein AM), P**7** exhibited higher sensitivity (Figure 5h,j), better photostability (Figure 5k), biocompatibility (Figure 5l), and enhanced tracking ability (Figure 5m), which are owing to the synergistic effect and the high molecular weight of the polymer.

**FIGURE 6:**
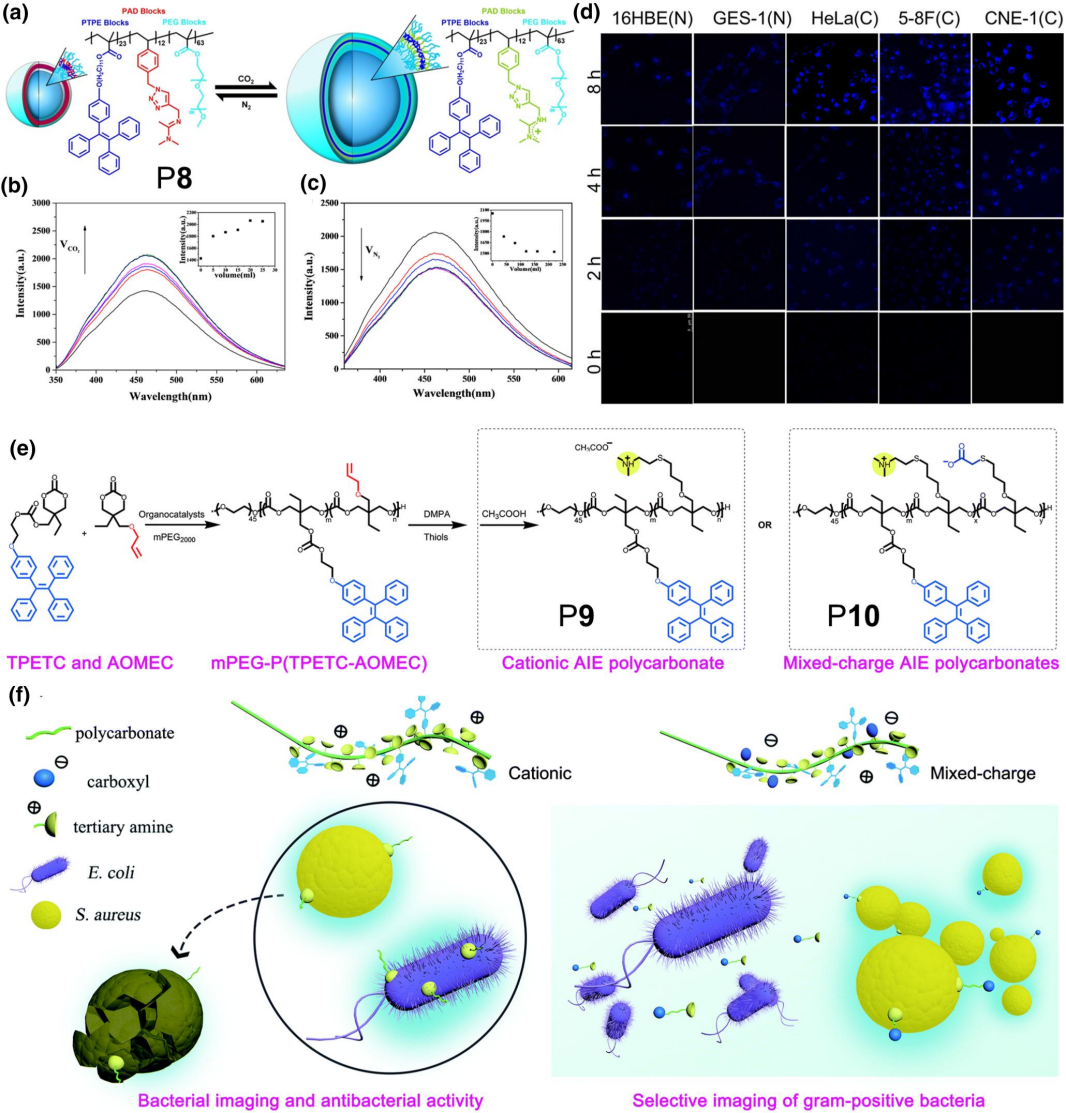
(a) Schematic diagram of the recognition process of P**8**. (b) PL spectra of P**8** aqueous solution at different volumes of CO_2_. (c) PL spectra of CO_2_‐treated P**8** aqueous solution at different volumes of N_2_. (d) CLSM images of 16HBE human bronchial epithelial cells (normal), GES‐1 human gastric mucosa epithelial cells (normal), HeLa cells (cancer), 5–8F nasopharynx cells (cancer), and CNE1 nasopharynx cells (cancer) after incubation with polymer P8 (1 mg mL^−1^) for 0, 2, 4, and 8 h, respectively. Reproduced with permission: Copyright 2019, American Chemical Society.^[25]^ (e) Synthesis routes of P**9** and P**10**. (f) Schematic diagram of P**9** and P**10** for bacterial imaging and killing. Reproduced with permission: Copyright 2021, Royal Society of Chemistry.^[26]^ CLSM, confocal laser scanning microscope; PL, photoluminescence.

Carbon dioxide (CO_2_) is an important metabolite in the process of aerobic respiration of cells. In contrast to normal cells, cancer cells have a higher metabolic rate, which means a higher rate of CO_2_ production. This difference provides a powerful assistance in cancer diagnosis. Wang and co‐workers prepared a novel “breathable” block polymer P**8** based on reversible addition‐fragmentation chain transfer polymerization (Figure 6a).^[25]^ The polymer P**8** consisted of an AIE unit, an amidine‐containing CO_2_‐responsive unit and a hydrophilic unit, and negligible emission could be observed in aqueous solution. With the increase of CO_2_ concentration in the solution, the hydrophobicity of the P**8** increased significantly due to protonation, which led to the motion restriction of the AIE units and eventually enhanced fluorescence. Interestingly, when increasing the nitrogen (N_2_) concentration in the CO_2_‐treated P**8** aqueous solution, that was, deprotonation, the fluorescence subsequently weakened (Figure 6b,c). As shown in Figure 6d, the fluorescence intensity of P**8** was significantly higher in tumor cells than that in normal cells, which provided an idea candidate for distinguishing tumor tissue from normal tissue.

Identifying the type of bacteria is of crucial importance in the treatment of clinical infections. Compared with standard plate colony counting and polymerase chain reaction, using fluorescence imaging to identify the type of bacteria is time‐saving, operator‐friendly and cost‐effective. In 2021, Lang and co‐workers designed and prepared a polycarbonate which contained three main parts, a polyethylene glycol (PEG) backbone, an AIE unit side chain and an alkene side chain, which could be used for post‐modification (Figure 6e).^[26]^ The antimicrobial properties and selective imaging ability of polymers P**9** (cationic AIE polycarbonate) and P**10** (mixed‐charge AIE polycarbonate) could be obtained by thiol‐ene post‐modification reaction. Due to the strong positive charge of the cationic polymer P**9**, it could effectively stain gram‐positive and gram‐negative bacteria. Interestingly, with the increase of bacterial concentration, the PL intensity of the polymer solution was subsequently increased, which was attributed to the introduction of AIE units. As for P**10**, a mixed‐charge polymer, selective imaging of gram‐positive bacteria could be achieved at a relatively wide pH range due to the presence of the isoelectric point (Figure 6f).

**FIGURE 7:**
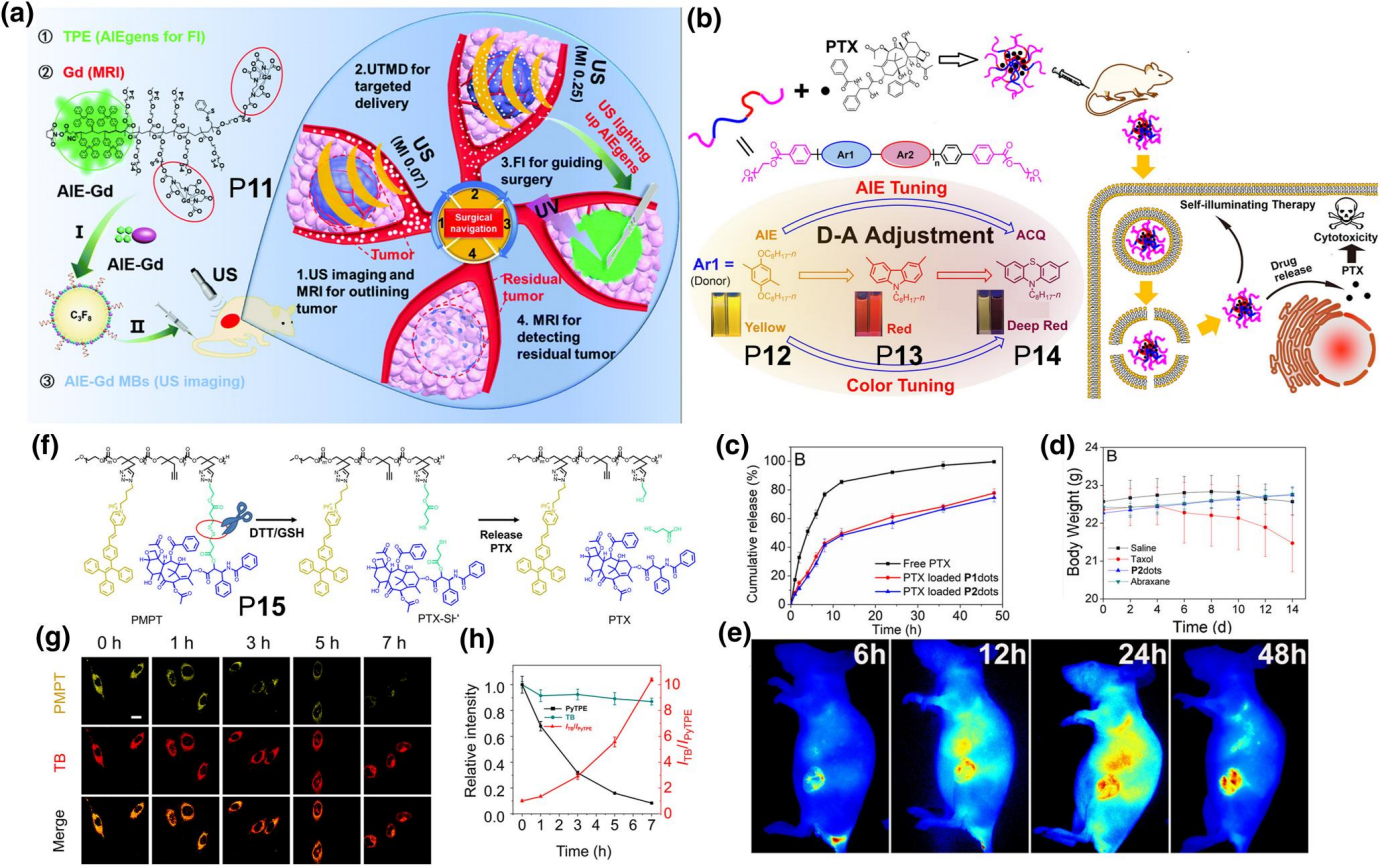
(a) Schematic diagram of the mechanism for lighting up P**11** microbubbles during surgical navigation through ultrasound targeted microbubble destruction. Reproduced with permission: Copyright 2021, Royal Society of Chemistry.^[27]^ (b) Structures of polymers P**12–**P**14** and schematic diagram of using AIE polymers as drug carriers for cancer therapy. (c) Cumulative release profiles of free PTX and PTX‐loaded polymers in PBS over 48 h. (d) Body weight of mice after different treatments during cancer treatment. (e) *In vivo* fluorescence imaging of MCF‐7 tumor xenograft mice at 6, 12, 24 and 48 h after intravenous injection of DiR‐loaded polymer. Reproduced with permission: Copyright 2019, American Chemical Society.^[28]^ (f) Schematic diagram of the release mechanism of PTX in P**15** under reductive environment. (g) CLSM images of HeLa cells in different channels after incubated with P**15** for 2 h. (h) Quantitative analysis of intracellular fluorescence intensity changes. Reproduced with permission: Copyright 2021, American Chemical Society.^[29]^ AIE, aggregation‐induced emission; CLSM, confocal laser scanning microscope; PTX, paclitaxel.

Compared with single imaging technique, multimodal diagnosis and treatment have obvious superiority in spatial/temporal resolution and imaging sensitivity, and is more suitable for different stages of disease treatment. As a proof of concept, Zha and co‐workers prepared an ultrasound‐responsive smart AIE polymer P**11** with tri‐modal imaging potential (Figure 7a).^[27]^ With the capabilities of ultrasound imaging, magnetic resonance imaging and AIE unit‐based fluorescence imaging, the P**11** could be used for different periods of surgical treatment.

Traditional drug carriers, such as PEG and liposomes, are difficult to study the distribution of drugs *in vivo* because they have no tracing capability. The introduction of fluorescent low‐mass molecules can solve this problem, but its biosafety, instability and leakage problems still need to be considered. More importantly, non‐AIEgen loaded in nanoparticles will encounter the problem of low PL quantum yields due to the ACQ effect. Notably, AIE polymers present distinct advantages in drug delivery, tracing and biosafety. In 2019, Hua, Yuan and co‐workers constructed three amphiphilic polymers P**12–**P**14** by simple coupling reactions (Figure 7b).^[28]^ Interestingly, the transformation of polymers from ACQ to AIE could be achieved by changing the electron‐donating groups in the polymers. Owing to the introduction of AIE units and the interaction of conjugated structures, after loading the cancer chemotherapy drug paclitaxel (PTX), AIE polymers exhibited anti‐drug leakage ability (Figure 7c), desirable biosafety (Figure 7d), good tracing properties (Figure 7e) and outstanding anti‐tumor properties. Meanwhile, the AIE polymer drug delivery system showed better biocompatibility and anti‐tumor effect than commercial drug delivery system of Abraxane, indicative of its bright future in drug delivery.

Compared with encapsulation, drug delivery via dynamic covalent bonding is more effective for the enrichment and release of drug molecules, as well as improving its biosafety. Meanwhile, chemotherapy‐based combination therapy is more effective than single therapy. Lou and co‐workers constructed a self‐guiding smart AIE polymeric micelle P**15**, including a reduction‐sensitive PTX prodrug and two AIE photosensitizers (Figure 7f).^[29]^ The AIEgens and PTX prodrugs located in the side chains to enhance the hydrophobicity of the polymer, which allowed the polymer to exist as micelles before reaching the destination. Interestingly, when P**15** entered tumor cells as aggregates, it has a clear fluorescence signal because of the AIE property, but with high concentration of glutathione leading to disulfide bond breakage and PTX releasement, the fluorescence gradually disappeared. The separation of PTX increased the hydrophilicity of the polymer, making it more difficult to exist in the aggregate form. In addition, by combining this feature, P**15** and additional encapsulated AIE low‐mass molecules could achieve the ratiometric fluorescence imaging of drug release process (Figure 7g,h).

### 2.3 Synergistic amplification

Low dosage of drugs with high therapeutic effect is a key goal in disease treatment. The advantage of AIE polymers over low‐mass AIEgens is an attractive issue, which has been demonstrated in several systems. For example, compared to low‐mass AIE molecular analogs, AIE conjugated polymers possess larger molar absorbance coefficients and more energy levels in each energy band, indicative of a better ability for light harvesting and energy transfer. These unique properties can be utilized for bioimaging and disease therapy.

**FIGURE 8:**
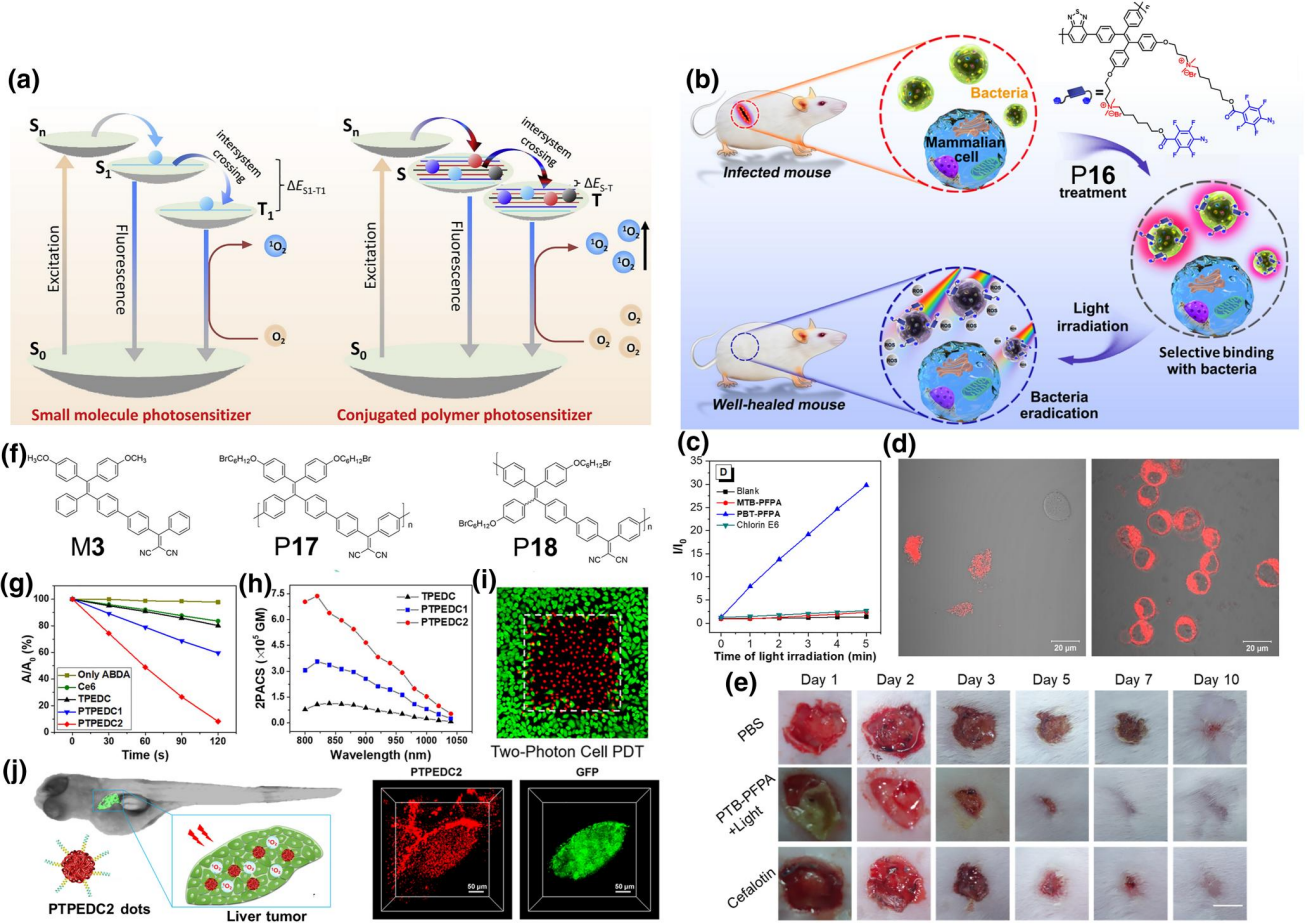
(a) Schematic diagram of the working mechanism and design guidelines of polymerization‐enhanced photosensitization. Reproduced with permission: Copyright 2018, Elsevier.^[30]^ (b) Chemical structure of P**16** and schematic diagram of selective antibacterial behaviors. (c) ROS generation capacity of polymer P**16**, low‐mass analogs and Ce6 under white light irradiation (10 mW cm^−2^). (d) CLSM images of HeLa cells and mixed sample (HeLa cells + microorganisms) after incubated with P**16** or low‐mass analogs. (e) Photographs of the *S. aureus*‐infected skin of mice during treatment with the different formulations. Reproduced with permission: Copyright 2020, John Wiley and Sons.^[31]^ (f) The chemical structures of M**3**, P**17** and P**18**. (g) The ^1^O_2_ generation capacity of M**3**, P**17**, P**18** and Ce6 under white light irradiation. (h) Two‐photon absorption cross‐section spectra of M**3**, P**17** and P**18**, respectively. (i) The precise two‐photon PDT based on polymer P**18**. (j) Schematic diagram of two‐photon imaging and PDT in a zebrafish liver tumor model. Reproduced with permission: Copyright 2019, American Chemical Society.^[32]^ CLSM, confocal laser scanning microscope; PDT, photodynamic therapy; ROS, reactive oxygen species.

Photodynamic therapy (PDT) is a promising approach for disease treatment. Under light irradiation, photosensitizers can produce ROS efficiently, which can be used to inactivate the surrounding cancer cells and bacteria. For low‐mass AIE molecules, the ROS generation ability can be promoted by structural modulation, such as introducing heavy atoms and reducing the gap of energy levels between the lowest singlet level (S_1_) and the lowest triplet level (T_1_) (Δ*E*_S1‐T1_). These methods are used to enhance intersystem crossing (ISC) process, but the consequent dark toxicity and weakening of the light absorption capacity outweigh the benefits. In contrast to traditional photosensitizers, AIE polymers exhibit enhanced fluorescence and ROS production in the aggregate state. At the same time, the repetitive conjugated structure in polymers further enhances the ability of ROS production. With these excellent properties, AIE polymers are the excellent candidates for PDT applications. In 2018, Liu and co‐workers proposed the concept of polymerization‐enhanced photosensitization to guide the synthesis of highly ROS‐producing photosensitizers (Figure 8a).^[30]^ The experimental results and theoretical calculation confirmed their opinion that the increase of conjugation length made the light absorption ability and ISC process of the polymer enhanced, which were more beneficial to the ROS production.

The ROS produced by photosensitizers can be used for efficient bactericidal applications without drug resistance. However, many PDT systems are limited by poor ROS generation ability in aggregate state and low selectivity. AIE polymeric photosensitizers with outstanding ROS production effect and modifiable side‐chain are a good choice. Qin, Tang and co‐workers designed and synthesized a smart AIE polymer P**16** which could generate ROS and free radicals upon light irradiation (Figure 8b).^[31]^ The introduction of the D‐π‐A structure in the main chain guaranteed the ability of the polymer to generate ROS efficiently. Compared with the low‐mass photosensitizer Chlorin E6 (Ce6) and its low mass analogs, P**16** exhibited a significant ROS generation capacity in PBS solution (Figure 8c). Interestingly, by introducing quaternary ammonium group in the middle of the side chains, the smart AIE polymer P**16** could achieve differentiation between mammalian cells and microbes, and selective binding with the latter (Figure 8d), which is of great clinical value. As a sharp contrast, the corresponding analogs didn't have selectivity, which exhibits the advantage of polymers. The selectivity was due to the fact that the positively charged side chains of P**16** prefer to bind with microbes via electrostatic interaction, while the balanced hydrophilicity and hydrophobicity of the polymer prohibited the binding with mammalian cells. The *S. aureus* skin infection models demonstrated that P**16**‐based PDT treatment could inhibit bacterial infection and promoted wound recovery more effectively than the clinical drug of cefalotin (Figure 8e). This work provides an important reference for the preparation of AIE polymeric antimicrobial agents with high biocompatibility and ROS generation efficiency, which is expected to be used for clinical applications.

According to the mechanism of ROS generation by one‐photon excited PDT, materials with visible light absorption are easier to generate ROS, regardless of polymers or low‐mass molecules. However, most of the clinically available photosensitizer absorption is in the visible light region with limited tissue penetration depth, which restricts the application of PDT *in vivo*. Compared with single‐photon excitation, two‐photon excited PDT (2PE‐PDT) can significantly increase the penetration depth of light and improve the treatment effect, which aroused tremendous interests among researchers. However, for low‐mass molecules, having both large two‐photon absorption (2PA) cross section and high ROS generation capacity are contradictory, which is difficult to achieve. In order to solve this problem, Liu and co‐workers demonstrated that the efficiency of two‐photon photosensitization can be enhanced by polymerization.^[32]^ The AIE polymers P**17** and P**18** were designed and prepared by using low‐mass AIE unit M**3** with high ROS production capacity (Figure 8f). The results of ROS generation efficiency experiments and wavelength‐dependent two‐photon cross section illustrated that the ROS generation capacity and 2PA cross section of the photosensitizer could be significantly improved by polymerization (Figure 8g,h). Notably, compared with P**17**, P**18** with longer conjugate length exhibited superior ROS generation efficiency and 2PA cross section, which was also consistent with the results of time‐dependent density functional theory studies. As shown in Figure 8i, thanks to the accuracy of the high‐intensity focused pulsed laser, 2PE‐PDT could achieve precise killing of cancer cells in selected areas, which had important reference value for the precise treatment of local important organs. Not coincidentally, Tang and co‐workers reported a similar phenomenon, illustrating that the photosensitization ability of the low‐mass molecules can be significantly improved by polymerization.^[33]^

Surgery with fluorescent navigation facilitates the removal of tumors with high accuracy. However, fluorescence signals are usually influenced by the biological microenvironment, such as the autofluorescence of cells and biological tissues, which limits the accuracy and resolution of fluorescence imaging.

**FIGURE 9:**
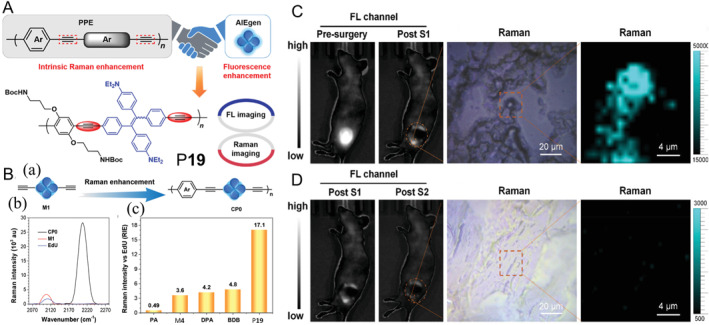
(A) Schematic diagram of the design strategy for functional AIE polymer P**19**. (B) (a) Schematic diagram of the polymerization‐induced Raman enhancement from M**4** to P**19**. (b) Raman spectra and (c) Relative Raman intensity versus EdU of phenylacetylene, M**4**, diphenylacetylene, buta‐1,3‐diyn‐1‐ylbenzene and P**19**. (C) Fluorescence and Raman imaging of P**19** treated tumor‐bearing mice before and after S1 treatment. (D) Fluorescence and Raman imaging of P**19** treated tumor‐bearing mice before and after S2 treatment. S1 and S2 treatment refer to the first and second fluorescence and Raman dual‐modality fluorescence‐guided tumor resection surgery, respectively. Reproduced with permission: Copyright 2021, John Wiley and Sons.^[34]^ AIE, aggregation‐induced emission; EdU, 5‐ethynyl‐2′‐deoxyuridine.

Although a large number of near‐infrared fluorescent materials have been reported, including low‐mass molecules and polymers, to conquer this problem, a single imaging modality has never been the most ideal answer. Raman imaging with stable cell‐silent regions (1800–2800 cm^−1^) combined with fluorescence imaging is expected to meet the demands. In 2021, Tang and co‐workers proposed a “win‐win” strategy for surgical navigation based on fluorescence imaging combined with Raman imaging (FLI‐RI).^[34]^ As shown in Figure 9A, ethynyl groups were introduced into polymer P**19** as the main source of Raman signal, which made it a good candidate for FLI‐RI. Remarkably, compared with its lower mass analogs, P**19** exhibited higher Raman signal intensity, which was attributed to the molecular wire effect of polymer, resulting in Raman signal amplification (Figure 9B). Furthermore, compared wiht 5‐ethynyl‐2′‐deoxyuridine (EdU, a standard alkyne Raman tag), diphenylacetylene (DPA, conjugated acetylene) and buta‐1,3‐diyn‐1‐ylbenzene (BDB, diacetylene derivative), P**19** showed ultra‐high Raman signal intensity, making it highly promising for Raman imaging. It was difficult to completely remove the tumor by surgery with the help of fluorescent navigation only (Figure 9C). However, after secondary surgery with the aid of Raman imaging, the signal of residual tumor couldn't be detected under fluorescent imaging, which meant FLI‐RI could achieve precise differentiation of tumor boundary from normal tissue to achieve precise removal of tumor (Figure 9D). This strategy for preparing AIE conjugated polymers with polymerization enhanced Raman signals provides an insight into the development of FLI‐RI dual‐modal imaging agents for surgery navigation.

## 3 CONCLUSIONS AND FUTURE PERSPECTIVES

Thanks to the continuous efforts, researchers have developed a great number of novel AIE polymers with advanced structures and multifunctional properties, which are widely used in biosensing, imaging and therapy, etc. In this review, we discussed the preparation of novel AIE polymers and, briefly summarized the unique multifunctional integration properties and synergistic effect of smart AIE polymers for biomedical applications in recent years.

In particular, we emphasize the advantages of smart AIE polymers with the properties over the related low‐mass analogs and commercial probes. The advantages of smart AIE polymers can be briefly summarized as follows:

1) When the AIE units are integrated in the side chain of polymers, the original properties of the polymer backbone will be retained, such as good substrate for biomolecule synthesis, and temperature responsive properties, which is beneficial for the construction of multifunctional polymers. Meanwhile, the AIE units can significantly change the hydrophobic property of the polymers, and the optical performance can be turned and enhanced by using AIE property. Importantly, these functions are difficult to be achieved by the related low‐mass analogs.
2) When the AIE units located in the polymer backbone, especially D‐A or D‐π‐A type conjugated polymers, the strong conjugated structure can offer the polymers with good photo‐stability, regulated photosensitization capability and desirable fluorescence intensity. This is attributed to the following two reasons: the strong D‐A conjugated structure can significantly reduce the Δ*E*_ST_, which is beneficial for ISC process and generating more ROS. Meanwhile, due to the D‐A structure, the separation of the highest‐energy occupied molecular orbital (HOMO) and the lowest‐energy unoccupied molecular orbital (LUMO) makes it possible for the polymers to have longer absorption and emission wavelength, which is more desirable for *in vivo* bioimaging. On the other hand, the extended conjugated backbone allows the polymer to have more energy levels in the same energy band, which also promotes the ISC process resulting in more ROS production.

Over the past two decades, AIE polymers have achieved remarkable progress in biomedical science. It is believed that with further research, more and more impossibilities in this field will become possible. Meanwhile, the following aspects might need more effort to focus on. First, continuous efforts are needed to develop efficient and green synthetic strategies for the preparation of novel AIE polymers, especially metal‐free and even spontaneous polymerizations. The *in situ* preparation strategy of AIE polymers, in other words, lab in cell, is an interesting direction. In particular, the selection and design of monomers are worth to thinking deeply. Second, the AIE polymers with good ROS generation capability were obtained at the expense of its PL quantum efficiency. More structures or strategies should be designed or proposed to achieve synergistic enhancement of ROS generation efficiency and PL quantum efficiency, which will provide more opportunities for AIE polymers in biomedical applications. Last but not least, it is difficult to express all the opportunities and challenges here. With this review, we hope to demonstrate the advantages of AIE polymers and encourage more researchers to focus on AIE polymers and explore their broad potential.

## CONFLICT OF INTEREST STATEMENT

The authors declare no conflict of interest.

## Data Availability

Data sharing not applicable to this article as no datasets were generated or analyzed during the current study.
